# Comparison of Effects of Propofol and Isosorbide Dinitrate during Rewarming on Cardiopulmonary Bypass

**DOI:** 10.12669/pjms.324.10190

**Published:** 2016

**Authors:** Aamir Furqan, Sohail Ahmad, Liaqat Ali, Rahat Akhtar, Mr. Mirza Ahmad Raza Baig, Rana Altaf

**Affiliations:** 1 Dr. Aamir Furqan, FCPS. Assistant Professor, Department of Anesthesia, Multan Institute of Kidney Diseases, Multan - Pakistan; 2 Dr. Sohail Ahmad, FCPS. M.Sc Pain Medicine, Assistant Professor of Anesthesia, Ch. Pervaiz Elahi Institute of Cardiology, Multan, Pakistan; 3 Dr. Liaqat Ali, FCPS. Assistant Professor of Anesthesia, Multan Medical and Dental College, Multan, Pakistan; 4 Dr. Rahat Akhtar, MBBS. Woman Medical Officer, Nishter Hospital Multan, Pakistan; 5 Mr. Mirza Ahmad Raza Baig, B.Sc Hons. Cardiac Perfusion. Clinical Perfusionist; 6 Prof. Rana Altaf, FCPS, FICS, Professor of Anesthesia, Chief Anesthetist, Ch. Pervaiz Elahi Institute of Cardiology, Multan, Pakistan

**Keywords:** Cardiopulmonary Bypass, Isosorbide Dinitrate, Propofol

## Abstract

**Objectives:**

Comparison of effects of propofol and isosorbide dinitrate during rewarming on cardiopulmonary bypass in patients undergoing coronary artery bypasses grafting.

**Methods:**

It was randomized prospective clinical trial. One hundred and twenty patient (120) undergoing CABG surgery were included in this study. Group-I (Study group, n=60): in which only propofol infusion used during rewarming and Group-II (control Group, n=60) in which isosorbide dinitrate and propofol infusion combination was used during rewarming. The data was entered and analyzed through SPSS Version 19. Independent sample T-test and chi-square test were used for data analysis. P value of ≤ 0.05 was taken as significant.

**Results:**

Mean arterial pressures during rewarming were 63.41±3.61 mmHg in propofol group versus 60.80±4.86 mmHg in control group (p-value 0.001). Core temperature on weaning from cardiopulmonary bypass was 37.11±0.49 °C in propofol group and 37.00±0.18 °C in control group. After drop in core temperature was little more in propofol group (1.02±0.36 °C) versus 0.96±0.37 °C in control group but this difference was not statistically significant (p-value 0.41). Mean Ventilation time after surgery in propofol group was 4.65±0.65 hours versus 5.03±0.81 hours in control group (p-value 0.006).

**Conclusion:**

Propofol alone is capable of fulfilling the requirements of adequate rewarming during Cardiopulmonary bypass and can produce more hemodynamic stability and early post-operative recovery.

## INTRODUCTION

Whole body hypothermia is a routinely used method to protect vital organs during cardiac surgery. Even mild degree of hypothermia is thought to protect against cerebral ischemia and myocardial damage during cardiac surgery.^1^,^2^ During surgery the body is cooled to about 28-30°C by means of hypothermia and heart lung machine. When the surgery is about to complete, the body is rewarmed with the help of hypothermia and heart-lung machine. During rewarming, the core body parts (head and trunk) are rewarmed more rapidly to normal temperature values as compared to the peripheral body parts.^3^ After weaning the patient from cardiopulmonary bypass the body is allowed to self-equilibrate its temperature and the heat redistribution is circulated from warmer parts of the body to the inadequately warmed peripheral body parts.^4^,^5^ This causes a decrease in temperature of the core organs (afterdrop).^6^,^7^ The resultant hypothermia may trigger shivering^8^ and can increase the risk of other complications e.g. inhibition of coagulation, reduction in resistance to surgical site infections and increase in myocardial stress.^9^-^12^ Several methods have been used to reduce the incidence of afterdrop such as local rewarming of peripheral tissues during rewarming of the patient. Some studies have shown that Forced-air blankets can help to prevent afterdrop.^3^,^13^

The second approach to prevent afterdrop is to induce pharmacologic vasodilatation during rewarming that will facilitate heat transfer from core to peripheral tissues.^14^ This method was 1^st^ used by Noback et al., in 1980.^15^ Later on some researchers concluded that the magnitude of afterdrop is reduced when sodium nitropruside was used to induce vasodilatation during rewarming phase of cardiopulmonary bypass.^16^,^17^ In these studies the patients were cooled to about 28 °C and pump flows during rewarming were maintained nearly about 2.4 to 2.6 l/min/m^2^. Now a days, isosorbide dinatrate is routinely used for proper rewarming during cardiopulmonary byass because of its vasodilatory effects.

Propofol a potent anaesthesia induction agent is routinely used in cardiac surgery. Propofol also has additional benefits of cerebral and myocardial protection through mechanism of reduction of oxygen consumption.^18^ Propofol shows its vasodilating effect both on veins and arteries through Ca^+^ channel blocking properties. Propofol also has vasodilating effect on coronary arteries through this same Ca^+^ channel blocking properties.^19^ So propofol induced vasodilation during rewarming phase of cardiopulmonary bypass can ensure proper rewarming. Therefore we conducted this study to compare vasodilating effect of isosorbide dinitrate and propofol during rewarming on cardiopulmonary bypass. Because if propofol has same effect when used alone then this will be very beneficial for patient management during and after cardiopulmonary bypass and there will be a less drug exposure.

## METHODS

It was randomized prospective clinical trial. Ethical approval was taken from the Department of Academic Affairs of Ch. Pervaiz Elahi institute of cardiology before starting the research work. One hundred and twenty (120) patients undergoing CABG surgery were included in this study. The patients were randomly assigned into two equal groups using a binary number generator system. Group I (Study group): in which only propofol infusion was used during rewarming and Group II (control Group) in which isosorbide dinitrate and propofol infusions combination was used during rewarming phase of cardiopulmonary bypass. All patients undergoing cardiopulmonary bypass having duration of cardiopulmonary bypass between 90-120 minutes and lowest temperature of about 28-30 °C during surgery were included in this study. Patients having ejection fraction <40%, who required inotropic support before going on bypass, lowest temperature <28 °C during the procedure, having contraindication to propofol or isosorbide dinitrate were excluded.

In both groups, peripheral venous cannulas and radial artery cannulas were inserted under general anesthesia. Anesthesia induction was achieved using fentanyl (3-5 µg kg^-1^), midazolam (0.02-0.05 mg kg^-1^), and propofol (1-2 mg/kg) and atracurium bromide (0.5 mg kg^-1^). Propofol infusion was started at a rate of 25 µg/kg/min and continued before, during and after termination of cardiopulmonary bypass in both groups. Tracheal intubation was done, and mechanical ventilation using air and oxygen was started to maintain normocapnia. After that central venus catheter (CVP) was inserted in the right internal jugular vein in all patients.

Cardiopulmonary bypass circuit was primed using ringer lactate solution 1500 ml, mannitol 100 ml and 40 ml of sodium bicarbonate. In all patients venous cannulation was achieved using a two-stage venus cannula, and for arterial cannulation a straight tip ascending aortic cannula was inserted into the ascending aorta. Immediately after going on bypass, atracurium bromide and fentanyl was given by the Perfusionist to maintain their normal levels in blood due to hemodilatory effects of cardiopulmonary bypass circuit. Blood flow during cooling was maintained at 2.4 l/min/m^2^ in all patients. The patients were cooled to about 30-28 °C. After achieving the target temperature blood flow was reduced to about 2.0 l/mim/m^2^ at 30 °C and 1.8 l/min/m^2^ at 28 °C. The heater cooler unit used for cooling and rewarming of the patients was Maquet HCU40 heater-cooler unit (Maquet Holding B.V. & Co. KG Kehler Strasse, Rastatt Germany). At the start of rewarming isosorbide dinitrate infusion was started along with propofol in control group. And only propofol infusion was continued in study group. Blood flow during rewarming was maintained at the rate of about 2.4-2.6 l/min/m^2^ in every patient. Isosorbide dinitrate infusion at rate of 0.2 Ug/kg/minutes was started when patient was rewarmed at peripheral temperature to 32 °C in control group. The patients were rewarmed by maintaining the arterio-venous blood temperature gradient to not more than 3 °C. The bypass was discontinued at a mean nasopharyngeal temperature of 37 °C. Skin temperature was measured at four sites; upper arm, chest wall, thigh and leg. Mean skin temperature was calculated using a weighted equation described by Ramanathan.^20^

**Table-I:** Comparison of Environmental Characteristics Hemodynamic Characteristics between the groups.

Variable	Propofol Group	Control Group	P-value
Number of Patients	60	60	
Age (Year)	52.83±10.42	53.77±9.10	0.602
Gender (N) male/female	40/20	38/22	0.70
BMI kg/m^2^	27.43±4.7	26.38±3.7	0.18
OR temperature (°C)	23.58±0.92	23.61±0.83	0.83
ICU Temperature (°C)	24.50±0.81	24.50±0.81	1.00
Bypass time (min)	105.12±10.59	103.88±8.91	0.49
Mean Arterial Pressures during rewarming (mmHg)	63.41±3.61	60.80±4.86	0.001
Ventilation time after surgery (hours)	4.65±0.65	5.03±0.81	0.006

Quantitative Variables are presented as mean±SD, OR= Operating Room, ICU= Intensive Care Unit.

Mean Skin temperature: 0.3(upper arm + chest wall) + 0.2(thigh + leg)

The nasopharyngeal temperature and skin temperature were monitored continuously after discontinuation of Cardiopulmonary bypass until the core temperature stopped decreasing.

The collected data was entered and analyzed through SPSS version 19. Mean & standard deviation were used to present quantitative variables. Frequency and proportion were calculated for gender. Independent sample T-test and chi-square test were used for data analysis. P value of ≤ 0.05 was taken as significant.

**Table-II:** Comparison of Rewarming Characteristics between the groups.

Variable	Propofol Group	Control Group	P-value
Rewarming time (minutes)	48.58±6.46	47.38±5.72	0.28
Core temperature at Weaning off (°C)	37.11±0.49	37.00±0.18	0.10
MST at weaning off (°C)	34.67±1.38	34.7±1.41	0.87
Afterdrop in core temperature (°C)	1.02±0.36	0.96±0.37	0.41
MST at the end of afterdrop (°C)	35.01±1.04	35.10±1.37	0.82
Afterdrop Duration (°C)	35.8±5.19	34.2±5.02	0.09

MST= Mean Skin Temperature.

## RESULTS

The mean age of patients in this study was 52.83±10.42 years in propofol group and 53.77±9.10 years in control group. There was no significant difference in gender distribution and BMI of patients in this study. The environmental conditions regarding Temperature in Operating room (OR) and in Intensive Care Unit (ICU) were same between the two groups. Mean cardiopulmonary Bypass time and rewarming time were also same between the two groups.

Mean arterial pressures during rewarming were high in propofol group; in propofol group the mean arterial pressures were 63.41±3.61 mmHg versus 60.80±4.86 mmHg in control group (p-value 0.001). Core temperature on weaning from cardiopulmonary bypass was 37.11±0.49 °C in propofol group and 37.00±0.18 °C in control group. There was no significant difference in mean skin temperature on weaning from cardiopulmonary bypass. Afterdrop in core temperature was little more in propofol group (-1.02±0.36 °C) as compared to -0.96±0.37 °C in control group but this difference was not statistically significant (p-value 0.41). Mean duration of afterdrop was also little high in propofol group as compared to control group but with no significant difference (p-value 0.09). Mean skin temperatures at the end of afterdrop were also almost same between the two groups. We found earlier extubation in propofol group, mean ventilation time in propofol group was 4.65±0.65 hours in propofol group versus 5.03±0.81 hours in control group with highly significant p-value 0.006. We found good hemodynamic stability and early post-operative recovery in propofol group.

## DISCUSSION

Postoperative hypothermia is a common complication in patients undergoing hypothermic cardiopulmonary bypass than in patients undergoing other thoracic procedures not requiring the use of cardiopulmonary bypass (CPB).^21^ Blood flow to peripheral beds such as muscle and fat is reduced during hypothermic CPB because of intense vasoconstriction caused by hypothermia. During the rewarming phase, the peripheral parts rewarm slowly because of vasoconstriction and are still at hypothermic level when CPB is terminated at a normal core temperature.^22^ Afterdrop occurs as a result of redistribution of heat from warm core to cold periphery and due to heat loss from surgical incision sites to the environment.^4^,^5^ The primary factors responsible for afterdrop are; core to peripheral temperature gradient, cooling temperature, blood borne convection of heat and duration of rewarming.^14^

Pharmacologic vasodilation is a routinely used method to properly rewarm the patient during rewarming phase of cardiopulmonary bypass and hence to prevent afterdrop. Nitrates are widely used for uniform rewarming during CPB; a sudden fall in mean arterial pressures with wide fluctuations leading to the use of vasoactive drugs is a common problem with the use of nitrates.^23^ Propofol is widely used for anesthesia maintenance during cardiac surgery, it also has vasodilatory properties. When nitrates are used during rewarming, propofol infusion is also being used in these patients. So we conducted this study to see whether the use of propofol alone can result in adequate rewarming of the patient or not. Because if propofol results in adequate rewarming, the use of nitrates during rewarming can be discontinued. Hence, the requirement of vasoactive drugs will also be reduced.

In this study, we found no significant difference in MST at the time of weaning from CPB and at the end of afterdrop in propofol and control group. There was no significant drop in post-bypass temperature in propofol and control group. Rewarming time was also same between the two groups. But mean arterial pressures during rewarming were significantly high in propofol group as compared to control group (P-Value 0.001). Extubation time was also significantly less in propofol group as compared to the control group. Tuğrul M et al., also found higher extubation time when sodium nitropruside was used during the rewarming phase of CPB for proper rewarming.^16^ Chauhan P et al. concluded in their trial that use of nitroglycerine during rewarming along with propofol is associated with greater Afterdrop and higher volume requirements in the peri-operative period as compared to when propofol is used alone.^24^ In this study, we found that use of only propofol during rewarming is associated with uniform rewarming during cardiopulmonary bypass and early recovery in the post-operative period.

## CONCLUSION

Propofol alone is capable of fulfilling the requirements of adequate rewarming during Cardiopulmonary bypass and can produce more hemodynamic stability and early post-operative recovery.
